# Company size, work-home interference, and well-being of self-employed entrepreneurs

**DOI:** 10.1186/s13690-017-0243-3

**Published:** 2017-12-07

**Authors:** Isabelle Godin, Pierre Desmarez, Céline Mahieu

**Affiliations:** 1 School of Public Health, Université Libre de Bruxelles, 808, Route de Lennik, 1070 Bruxelles, Belgium; 2 Université Libre de Bruxelles, 50, Avenue F.D. Roosevelt, 1050 Bruxelles, Belgium

**Keywords:** Entrepreneurship, Very small enterprises, Work-home interference, Working conditions

## Abstract

**Background:**

The impact of working conditions on the health and well-being of workers of large enterprises has been widely described. This influence has not been studied as extensively in very small and medium-sized enterprises mainly due to methodological difficulties. Smaller organisations nevertheless constitute a reality that needs to be better understood.

**Methodology:**

The aim of this article is to better understand the working conditions of entrepreneurs in small and medium-sized enterprises, to describe the impact of these conditions on their health and well-being, and to learn how their work affects their private lives.

This is why a study was conducted in 2015–2016 on a selected sample of entrepreneurs in the Brussels-Capital Region (*n* = 140). The survey form included questions pertaining to the work environment, motivations underlying the choice of activities, robustness of the business, work-home interference, work-related stress, work satisfaction, self-reported health indicators, and socio-demographic status. The results were compared with those from another survey on workers in small shops conducted between 2012 and 2015 within the same Region (*n* = 104).

**Results:**

The number of entrepreneurs who participated in the survey added up to 140, with an even distribution between men and women. Two results are highlighted. The first concerns the difficulties faced by entrepreneurs working with a small team (1 to 4 employees): they are more stressed, report having heavy workloads, describe their health more negatively, consume more sedatives, and claim to suffer from loneliness more often than those working with larger teams or alone. Comparatively, in the study on shopkeepers, business owners working alone found themselves in a worse situation regarding their health and well-being.

The second finding involves the difficulties entrepreneurs face when it comes to combining work and family life, and for which gender inequalities were noted. This phenomenon remains insufficiently explored amongst small business owners.

**Conclusion:**

In spite of the difficulties encountered at work, commitment to their chosen profession remains strong amongst entrepreneurs. Our results enable us underscore the aspects of entrepreneurial activity that should be taken into account whilst setting up support mechanisms or promoting entrepreneurship, especially amongst and for women.

## Background

The impact of working conditions on the health and well-being of employees of large and medium-sized enterprises has been widely described, be it in Europe [1–12] or the United States or Asia [13–18]. Within the Belgian context, in particular, multiple longitudinal studies have documented this relationship in large enterprises [19–22].

Psychosocial risks (stress, imbalance between efforts and rewards, job insatisfaction, poor career opportunities…), work-home interference, workloads, and a leeway in the organisation of tasks appear to be the main factors that have a significant impact on the physical and mental health of employees [8, 10, 11, 14].

In very small and medium-sized enterprises, the situation is considerably less known for several reasons, with the most important being methodological difficulties. Large enterprises are more easily identifiable and their employees, clearly listed, are easier to contact. Workers can simply participate anonymously and confidentially in surveys. Moreover, they are often represented by organisations that call for better working conditions and support the carrying out of surveys.

In comparison, (very) small firms are more difficult to find, employee representation within them is relatively rare, the spatial proximity between colleagues, between employees and their superiors, the unconventional working hours, and the pace of work all impede workers from participating in surveys on working conditions.

While quantitative methods are most frequently used for large companies, as in the field of social epidemiology, they appear less suitable for microenterprises. On the one hand, this is because the terms and conditions of statistical methods are difficult to meet due to statistical inference, sample sizes, the representative nature of workers or sectors, great variability of job characteristics and of working conditions. The extremely diverse and often short-lived nature of these enterprises also adds to the difficulty. In order to avoid administrative obstacles, some enterprises choose to change their corporate name or their statute [23]. These factors complicate the sampling frame process. On the other hand, the data collection procedures per questionnaire (self-administered or face-to-face) are difficult to apply mainly for organisational and logistical reasons.

This is why qualitative or semi-qualitative methodologies are favoured in such contexts, often through an in-depth view of work realities, as described by Ekanem [24], but this approach restricts the possibilities for comparative evaluation with the results obtained from large enterprises.

Despite several authors having identified certain work specificities in very small and medium-sized enterprises [25] in terms of autonomy, number of hours worked, [26], work pressures, and the corresponding link with health [25], smaller organisations clearly constitute a reality that needs to be better understood and documented. Those particular working conditions are common to employers and their employees.

Findings on this topic differ. In Gunnarsson et al.’s study about very small-sized enterprises (agriculture, manufacturing, construction, catering, and other related services), workers rated their health poorly with more musculoskeletal disorders [26, 27]. According to a study undertaken in Denmark by Sorensen et al., employees of very small and medium-sized enterprises have greater risks related to their health and safety, but are nonetheless protected from psycho-social risks [28]. Other authors, for example Alvaga et al., observe better health outcomes amongst self-employed people in comparison with salaried employees [29].

(Very) Small-sized organisations generate an important number of jobs and are essential pillars of economic activity. Businesses with fewer than 10 employees represent 82% of all employers in Belgium, and jobs in Brussels are in companies employing fewer than 50 people [30], accordingly inviting the questions on the differences or similarities of the risks to health and well-being in small enterprises, knowing those in large-sized ones?

Therefore, the main aim of this article is to better understand working conditions of entrepreneurs and their relationship to work in very small enterprises, to describe the impact of these conditions on their health and well-being, and to learn how this can interfere with their private lives.

## Methods

### Studies’ design

A study conducted for the first time between 2012 and 2015 has already revealed the specificities of working conditions and their impact on the health and well-being of workers - employers and employees - in small shops and businesses in Brussels (retail and wholesale sectors, personal services, pharmacies, catering) [31, 32]. The methodology used involved a combination of in-depth interviews, self-administered questionnaires, and on-site observation, with the research being concentrated within a specific region (Brussels-Capital Region). Approximately 100 retailers (*n* = 104) from contrasting socio-economic areas were chosen and invited to voluntarily participate in the questionnaire part of the study.

A second study, also based in the Brussels region, was carried out in 2015–2016 by focussing on job characteristics of a set of entrepreneurs (self-employed). The methodology chosen included a survey made available online through the Brussels Enterprise Agency, Impulse.Brussels.^1^ This is a platform which offers support and guidance to entrepreneurs, be they first-timers or otherwise.

With the administration of the questionnaire being voluntary, and with no real census available for the above section of the population, it was neither possible to calculate the participation rate nor evaluate the representativeness of the participants. The anonymous questionnaire was available in both French and Dutch versions.

Since there is no existing list of entrepreneurs in Brussels, a convenience sample was constructed via the contact list of Impulse Brussels. All volunteer respondents with a completed form were included in the study.

Indicators and instruments.

The survey form included questions pertaining to the description of the work environment, motivations underlying the choice of activities, robustness of the business, work-home interference, sources of professional stress, work satisfaction, multiple health indicators (self-reported), and socio-demographic status.

Together with socio-demographic variables such as gender, age, and qualification, other factors such as career choice motivations, the impact of work on health (identified from a list of health problems caused by work itself), the consumption of sedatives, sleeping pills, the time dedicated to work, days off, subjective health assessment, and the financial health of the organisation commonly appeared in the database of retailers and entrepreneurs.

Kelloway’s questionnaire was utilised to measure work-home interference [33]. The questions determine the ways in which work impacts the quality of private life (work-home interference (W-H), 6 questions) and, conversely, how private life influences work (home-work interference (H-W), 6 questions). Based on these questions, two scores were composed, dichotomised with the 75th percentile as threshold, accordingly delineating the “high interference” category, in order to have a comparison point with previous studies [34, 35].

Chronic fatigue was evaluated through four questions proposed by Albert et al.’s chronic fatigue questionnaire from which a score was established [36]. The 75th percentile was once more used as the threshold for the “chronic fatigue” category.

Mow’s question on the centrality of work was utilised in order to ascertain the importance of work,^2^ [37]. This question is particularly relevant in a population of entrepreneurs, knowing the importance and the place taken by work in the life of self-employed.

The subjective health status was assessed through a close-ended question in five categories clubbed into two groups (“good/very good” and “average/(very) bad”).

### Analysis methods

The statistical analysis of the closed-questions was made in the form of frequency distribution, and when the application conditions were met, the differences between proportions were tested with Pearson’s chi-square test. The differences between averages were tested using the Student’s T-Test. The level of statistical significance was predetermined at 5%.

The data was systematically analysed according to gender, age, and size of the organisation (sole trader or worker, between 1 and 4 employees, more than four employees). A content analysis was conducted with respect to the open-ended questions.

Comparisons were carried out between working conditions and the health of these entrepreneurs with those of 104 small retailers who had previously participated in the 2012–2015 study.

## Results

### Description of participants

The number of participants in the study on entrepreneurs adds up to 140 with an even distribution between men and women. The study participants are relatively young, with a third being under 35 years of age and 27% aged between 35 and 44. Their level of education is rather high: 83% of the respondents have a post-graduate degree, with the percentage being only 36% amongst retailers.

### Description of businesses

The most represented sector is that of services (private or business): 50% of the businesses are involved in commercial activities (retail, wholesale, catering) whereas manufacturing (industry) accounts for 35%. The remaining 15% are businesses involved in the health, social, sports, or cultural sector.

The vast majority of entrepreneurs (70%) hold an independent status with the rest (30%) being salaried employees, potentially earning themselves a secondary, self-employed status. Very small-sized enterprises are predominant: 80% have fewer than five salaried staff, and in more than half of the cases, the entrepreneur works alone with the possibility of collaborating with a freelancer.

The firms in question are young: two-thirds amongst them have been in business for less than five years. Businesses with five or more employees are older than the others. They are also regarded as being less at risk of closing down in the near future (7% of these entrepreneurs believe they are likely to face closure while the figure lies at 27% for those who work alone and at 29% for businesses that employ between 1 and 4 employees, *p* < 0.05).

Overall, the financial situation of the businesses is assessed positively: close to two-thirds of the participants evaluate their firms as being stable or expanding. These figures are consistent with those from the study on small Brussels’ retailers wherein 65% of respondents estimated their businesses to be stable or growing.

There is, however, a slight distinction to be made. Firms with 1–4 employees were judged as being most at risk by their owners: 58% see their firm as being stable or growing as opposed to 61% who work alone and 85% who are surrounded by five or more employees (Table 1).

**Table 1:** Socioeconomic, demographic and enterprises’ characteristics (*N* = 140), Brussels entrepreneurs study 2015–2016, (*N* = 140)

Entrepreneurs	N	%	Enterprise	N	%
Sex (*n* = 137)			Sector (*n* = 135)		
Men	68	49,6	Services	68	50,4
Women	69	50,4	Trade/industry	48	35,6
Age (*n* = 123)			Health/sport/culture	19	14,1
<35 yrs	42	34,1	Enterprise exists since: (*n* = 136)		
35–44	33	26,8	In development phase	12	8,8
45 et+	48	39	0–3 yrs	61	44,9
Family situation			3–5 yrs	13	9,6
Couple	89	75,4	≥ 5 yrs	50	36,8
Not in couple	29	24,6	Number of coworkers (*n* = 135)		
Education			Works alone	71	52,6
≤ Secondary (1st level)	2	1,6	1–4	37	27,4
Secondary (2d level)	19	15,6	≥ 5	27	20
Post secondary	101	82,8	Financial stability (n = 135)		
Professional status			Stable	39	28,9
Self-employed	88	69,3	Growing	49	36,3
Wage worker (+ self employed)	39	30,7	Declining	9	6,7
			Dangerous situation	15	11,1
			Don’t know	23	17

### Motivation to work and the centrality of work

What were the reasons for the participants to be engaged in entrepreneurship? What are their prime motivations? What is the significance of work in their lives?

A list of nine factors relative to motivation (independence, salary, recognition, opportunities to develop and acquire new skills, etc.) was proposed to the participants to be ranked in descending order of the three primary motivators.

The quest for independence is cited amongst the two main incentives in 73% of the cases, 60% are concerned with creativity and 57% with personal development at work. In one-third of the cases, client relations figure in the list of prime motivations. The three main motivational factors are found to be the same across small retailers in Brussels, but client relations were more frequently cited by them than by the entrepreneurs.

Of the total responses provided by the 140 entrepreneurs (*N* = 459), the most common motivators concern autonomy (99 responses), creative opportunities (82 responses), and the development of personal skills (68 responses).

As a general rule, work is of great importance to the lives of these entrepreneurs: 20% chose the response “one of the most important aspects of my life”.

### Description of experience at work and workload management

More than half of the respondents (55%) report being “stressed” or “highly stressed” due to work. The primary sources (responses equal to 5 or 6 on a scale ranging from 1 [no real stress] to 6 [very high stress]) were revealed to be workloads (44%), liquidity problems (41%), and administrative burdens (39%).

Close to 4 in 10 entrepreneurs attest to having an “excessive” workload. They were asked if hiring extra personnel would ease this load, and if not, to explain their misgivings through a list of ten possibilities (hiring costs, fear of not being in control, struggles in finding personnel, etc.). More than half (57%) do not believe that hiring more workers would be a solution. They attribute their reasoning to the onerous nature of the task itself (first point cited), the difficulty in finding reliable staff (second point cited), or to the fact that the owners considers themselves to be the only ones who can do the job (third point cited).

The link between work-related stress and an excessive workload is clear: more than three-fourths of the respondents who consider their workload as heavy also describe themselves as being stressed, this figure being less than half for those who do not claim to have a heavy workload (*p* < 0.001).

The entrepreneurs affirm a near-constant connection to the workplace, be it a small proportion (22%) who claim to be able to “always” stop working at least one day a week, or those who have been unable to take a few days of leave over the course of the past 12 months (17%). Close to 30% of them acknowledge not having spent a single day offline, disconnected completely from their emails or professional phone calls in the past 12 months. Respondents in the shopkeepers’ study also had trouble distancing themselves entirely from their jobs: only 28% reported never having worked on Sundays – when shops are closed – and three-fourths admitted to regularly working past 6 p.m.

### Health and well-being

More than two-thirds of the respondents estimate their health to be “good” or “very good”. On a comparative age basis, these figures largely correspond to those of smaller retail workers in Brussels.

However, 36% regard their work as having a negative impact on their health, which is lower than those working in small shops (45%).

Stress, overall fatigue, and sleep disorders are most often cited as work-related health problems. The same complaints are found amongst shopkeepers but with added back pain. It should nevertheless be noted that 15% consider their work as having a positive influence on their health.

The link between the subjective health of the entrepreneurs and the association that they make between work and health is very clear: 57% who believe their work to have a negative impact on their health consider themselves to be in poor health as opposed to the 16% who do not associate their health with their professional activities (*p* < 0.001).

Twelve percent of entrepreneurs mention the use of sedatives or anti-depressants over the course of (past) four weeks, which is comparable to the figure reported by shopkeepers. Yet, the entrepreneurs are more likely to consume sleeping pills during the same period: 19% versus 14% for shopkeepers.

The chronic fatigue score shows no difference between both genders but is inversely proportional to age: the youngest have the highest score.

### Home work and work-home interference

There is a significant relationship between chronic fatigue and the interference between private and professional life, be it a reduction in the quality of private life due to work or vice versa - professional life impinging on private life (*p* < 0.05 and *p* < 0.001 respectively in Student’s T-Test).

Entrepreneurs who describe themselves as being stressed by their work have a statistically significantly higher work-home interference score than those who are not as stressed (*p* < 0.001).

By analysing the twelve different elements that make up the two scores, it is the difficulty in drawing a clear distinction between work and private life that is reported most often. 86% of respondents say they “often” or “always” think about work when they are at home.

The interference of work in private life is clearly more prevalent than private interference in professional life. However, there are substantial differences between both genders, with virtually all of the statements bearing a greater significance of work-home interference for men. They are particularly striking for the points "*My work puts me in a bad mood at home*" and "*I do not listen to what others say because I think about my work while at home*" (Table 2). The comparisons on the 2 summated subscales do not show any noticeable difference between men and women, hiding the important differences while analysing separately the 12 items.

**Table 2:** Work interference with private life, Brussels entrepreneurs study 2015–2016 (in %)

	Men	Women
After work, I have little energy to do what I have to at home (*n* = 137)	45.6	60.9
I think of work while at home (*n* = 136)	91.0	81.2
I do not listen to what others say because I think about my work while at home (*n* = 135)	32.4	16.4
I need to be alone for a while after work (*n* = 136)	49.2	39.1
My work puts me in a bad mood at home (n = 136)	11.8	5.9
I do not fully enjoy my private/family life due to my workload (*n* = 136)	49.2	44.9

The opposite is observed with regard to the impinging of private life on the quality of work life with the exception of two proposed statements. Only "I am tense and irritable at work because of what happens at home" and "My private/family life puts me in a bad mood at work" are reported more often by men than by women (Table 3).

**Table 3:** Private life interference with work, Brussels entrepreneurs study 2015–2016 entrepreneur study (in %)

	Men	Women
My working day is interrupted by private/family obligations (*n* = 135)	20.6	22.4
It is difficult for me to concentrate on work because of my private/family life (*n* = 135)	4.5	7.4
I am tense and irritable at work because of what happens at home (*n* = 135)	5.9	3.0
I am tired at work because of responsibilities related to my private/family life (*n* = 136)	10.4	14.5
I spend time at work thinking about things I have to do at home (*n* = 135)	14.7	16.4
My private/family life puts me in a bad mood at work (*n* = 135)	5.9	0

By comparison, in the study on shopkeepers, 27% felt it was difficult to combine work schedules with family life.

### Health, stress, and company size

In general, it is in situations where the entrepreneur works alone that his or her health indicators are the best.

It is in smaller businesses of 1 to 4 employees where the entrepreneur encounters the most difficulties in terms of health (negative subjective assessment, intake of sedatives), negative impact of work on health, and work-related stress compared to those who work alone or those from relatively larger companies (5 or more employees).

As for sources of stress at work, entrepreneurs with a small team (1 to 4 employees) stand out with a particularly high proportion of stress caused by administrative burdens (*p* < 0.05), production problems, client relations, and loneliness (*p* < 0.05). They are more likely to consider abandoning their activity than solo entrepreneurs or those involved in larger businesses.

More than 62% of these entrepreneurs identify a negative link between their work and health. This is the case for 39% of those who work alone or in larger companies or businesses (*p* < 0.05). In these organisations, workers are much more likely to carry over problems from their professional to their private life: amongst those with 1 to 4 employees, more than 41% have a level of work-home interference in the top quartile of the score,^3^ while the figure lies at 23% for those working alone (*p* < 0.01). More than 40% of them have only taken a maximum of 10 days off during the past 12 months (as opposed to 26% of workers in the other companies).

### Ambiguity of the relationship to work

In spite of the above-described difficulties encountered at work, commitment to their chosen profession remains strong amongst entrepreneurs: 85% do not contemplate ceasing their work or business activity, even when the company is in peril. More than 70% of them do not envision (or in very rare cases) halting their activities under such circumstances.

### What is course of action to be taken to improve entrepreneurship?

The respondents were asked to evaluate the usefulness of a dozen proposals put forward to improve their working conditions. A comment field was also available to them for a residual open-ended question.

The three propositions that garner the most interest are the introduction of sickness insurance for the business manager from the first day of illness (90% consider it “useful”), the availability of “training vouchers” to help cope with stress and burnout (77% consider it “useful”), and the lowering of labour costs to facilitate recruitment (77% consider it “useful”). There is a high demand for training, for instance, in effective delegation and management of work pressure (more than 70% positive responses).

In the suggestions spontaneously submitted by the entrepreneurs, it is the administrative, contractual, and legal aspects that are the first to be proposed (*“reducing the tax burden”, “facilitating recruitment”, “reducing notice periods”).* Several amongst them call for a sharing of experiences and more meetings between entrepreneurs (in the form of leisure or similar opportunities to swap work-related stories).

## Discussion

The defining characteristics of non-salaried workers, active in very small companies and businesses are not well known. Their situations and status vary, although several subgroups have a certain homogeneity in terms of working conditions. The aim of this article is to identify these conditions and their effects on health and quality of life by highlighting the particularities of entrepreneurs compared to workers in small shops within the same geographical context.

The solutions proposed in the literature for micro and small enterprises mainly concern the ergonomic or occupational safety issues, such as the WISE project implemented by the International Labour Organization (www/ilo.org/wise). Our study focuses more on the organisation of work and on the possibilities of developing structures that can support entrepreneurs and offer them concrete solutions.

Our respondents assess their health in a generally positive way much like those who participated in Gunnarrson’s study [26]. Their difficulties and sources of stress (workloads, administrative burden, employee management) are similar to those identified by Mairiaux et al. [38]. In accordance with other studies [39], our results show that entrepreneurs demonstrate a high level of commitment to their work and are often satisfied with it, especially because of the autonomy it provides. This commitment can, however, have certain negative consequences, such as an adverse impact on health or work-life balance. In this regard, two of our results need to be highlighted.

The first result concerns the difficulties faced by entrepreneurs working with a small team (fewer than five employees). Compared to entrepreneurs who work alone, with the occasional help of a freelancer, or those working with a larger team (at least five employees), the former find themselves in a more difficult situation. They maintain that they are stressed more often - mainly due to heavy administrative burdens and concerns about their business’ finances - and also claim to suffer from loneliness. Their workload is heavy and diverse - they are simultaneously entrepreneurs, employers and workers themselves. It is understandable that health and well-being do not feature in their list of priorities [23]. These results differ from those obtained from the study on shopkeepers. Amongst the latter, it was the business owners working alone who found themselves in the most precarious situation regarding their health or well-being**:** they experienced loneliness at the workplace, a lower well-being score**,** sleep disorders, or a lack of energy, for example.

After the launch of their business, small businesses owners find themselves at another critical stage of their company’s development when human resource and administrative management tasks are added to their responsibilities. These businesses can be identified as those requiring assistance (in this area) with respect to training or external support, for instance. Economically, these businesses create jobs during this development phase and must receive guidance so as to offer their employees high quality employment. Almost 30 years ago, Kets de Vries drew attention to similar difficulties faced by entrepreneurs in relation to administrative and team management tasks [40].

The second result to be highlighted in this study is the difficulty entrepreneurs face when it comes to combining work and family life. This phenomenon remains insufficiently explored amongst small business owners even though studies carried out in Belgium on employees of large companies have established the link between factors of stress at work, work-home interference and long-term absenteeism, anxiety, chronic fatigue, and negative subjective health assessment [34, 35]. Gender inequalities were also emphasized.

In the literature on groups close to that of this study, findings are rather varied [25]. The strong influence of work on the life of self-employed workers can nevertheless be explained by the blurring of boundaries between their work and private life [41]. Moreover, as Parasuraman and Simmers [42] have pointed out, entrepreneurs and freelancers emphasise their enormous freedom in terms of flexibility in the organisation of tasks and working hours. Yet, they find that once they are immersed in work duties, it is very difficult, especially for women, to maintain a work-life balance [42].

It should be noticed that at the European Level, working conditions of self-employed is receiving rising interest (data issued from the European Survey Eurofound) [43].

These two sets of results enable us to draw attention to two specific characteristics of entrepreneurial activity that should be taken into account whilst setting up support mechanisms or promoting entrepreneurship, especially amongst and for women.

On the one hand, while they are subject to strong expectations in terms of job creation, entrepreneurs, especially first-timers, strongly motivated by a desire for independence, find themselves in a critical phase. This is when they begin to surround themselves with a few employees. The administrative tasks weigh on them, which in turn has a negative impact on their health and eventually puts their independence or even the company at risk. On the other hand, their strong commitment to their professional duties, and the constant attention these require, often place entrepreneurs, especially women, in a situation where boundaries between private and professional life continue to blur.

### Scope and limitations

It was not possible to establish a sampling procedure in both studies given that the parent population was not known. It was, therefore, impossible to draw inferences from the data obtained and a selection bias cannot be excluded.

The possibilities for quantitative analysis were limited given the sample size. Requirements for the application of the statistical tests were not always met. However, even if the observed differences do not achieve the level of statistical significance, the comparisons and conclusions to be drawn can themselves be of interest.

The methods of collecting data through self-administered questionnaires in the two surveys are not identical and sections of the population concerned are also different. This in turn calls for adapted methodologies.

Notwithstanding the difficulties in approaching and studying this section of employees of all small businesses – a problem also touched upon in other publications – the combination of different methods made it possible to reach a relatively high number of these workers.

## Conclusion

This study is among the very few ones that could reach a population of self-employed workers, working in very small enterprises. The lack of registered data, the variety of their activities, their specific working conditions and tight schedules make such studies difficult to conduct. The results have to be understood in the framework of a descriptive and exploratory methodology.

In spite of the difficulties encountered at work, commitment to their chosen profession remains strong amongst entrepreneurs. Our results enable us underscore the aspects of entrepreneurial activity that should be taken into account whilst setting up support mechanisms or promoting entrepreneurship, especially amongst and for women. After the launch of their business, owners of smaller businesses are in a critical stage of their company’s development. Human resources and administrative management tasks are added to their responsibilities. The administrative tasks weigh on them, impacting negatively on their health and well-being. Their independence - or even the company- are eventually put at risk. Their businesses require assistance in terms of training or external support, as financial incentives, especially when they start their activity.

## Footnotes

1
http://www.abe-bao.be/en

2 “What is the importance of work in your life?”, scored on a scale of 1–7 with 1 being one of the least important things in life and 7 being one of the most important things in life.

3 Score summing up the workers’ responses through 12 scaled questions
